# Supplementary material for: Massively parallel characterization of transcriptional regulatory elements
Source: Nature. 2025 Jan 15;639(8054):411–20. doi: 10.1038/s41586-024-08430-9 (PMC11903340; doi:10.1038/s41586-024-08430-9)
Supplement: Supplementary file 1 — This files contents additional results about the biochemical features that predict regulatory activity and methods for motif-enrichment analysis, regression modelling, MPRAnn hyperparameter optimization, MPRALegNet architecture and data augmentation strategy and variant effect prediction. [file 41586_2024_8430_MOESM1_ESM.docx]

**SUPPLEMENTARY INFORMATION**

**Massively parallel characterization of transcriptional regulatory elements**

Vikram Agarwal, Fumitaka Inoue, Max Schubach, Dmitry Penzar, Beth K. Martin, Pyaree Mohan Dash, Pia Keukeleire, Zicong Zhang, Ajuni Sohota, Jingjing Zhao, Ilias Georgakopoulos-Soares, William S. Noble, Galip Gürkan Yardimci, Ivan V. Kulakovskiy, Martin Kircher, Jay Shendure, Nadav Ahituv

**SUPPLEMENTARY RESULTS**

**Biochemical features predict regulatory activity**

We set out to train regression models that can characterize regulatory activity using biochemical features. Previous work by our labs trained regression models to characterize enhancer activity, guided by the observation that the biochemical signals associated with the endogenous loci strongly explain lentiMPRA activity^10,14^. However, the data were limited in sample size (i.e., ~3,000 sequences) and underlying sampling bias (e.g., requiring elements to have a strong H3K27ac signal). Here, we took advantage of our sizable MPRA to comprehensively test which biochemical signals from the matched cell type explain cCRE activity (i.e., specifically, the subset of promoters and potential enhancers). We generated lasso regression models for each cell type based upon a set of 1,506 HepG2 biochemical descriptors; 1,206 K562 descriptors; and 277 WTC11/H1-ESC descriptors (**Supplementary Table 6**). These descriptors encompass features from DNase hypersensitivity, histone ChIP-seq, and TF ChIP-seq datasets. We used these biochemical experiments to compute a feature set by extracting signal intensities from genomic regions corresponding to our elements, and then averaging signals from the samples for the identical TF or histone mark in order to reduce feature redundancy. This led to a total of 655 HepG2 features, 447 K562 features, and 122 WTC11/H1-ESC features considered by the models. We were able to predict enhancer activities with high accuracy (Pearson r ≈ 0.72) in all three cell types (**Supplementary Fig. 5a**) using a 10-fold cross-validation (CV) approach on our data. Many of the top coefficients fit by these models correspond to DNase/ATAC-seq signal as well as ChIP-seq signal for transcriptional activators (e.g., HNF1A/HNF1B/HNF4A, NFYB/NFYC, SP1, YY1, and NRF1), coactivators (e.g., EP300 and KDM5A), repressors (REST, SUZ12, SIN3A, and HDAC1/2), chromatin organizers often found in insulators (CTCF and RAD21), and core transcriptional machinery proteins (POLR2G and TAF1) (**Supplementary Fig. 5b**). Despite these findings, model interpretation was inherently limited by the substantial degree of multicollinearity among features, as previously observed^14^. To minimize the chances that other important factors may have been overlooked by lasso regression, we identified alternative features which were highly correlated to the top-ranked features of our models (**Supplementary Fig. 5c-e**). This analysis revealed a large suite of TFs whose activity was strongly correlated to the top features, and identified other relevant components of cohesin, including SMC3 and STAG1 (**Supplementary Fig. 5c**). The variable feature count for each cell type led to the possibility of biasing performance. We thus benchmarked how a ‘universal’ feature set, merging features from all cell types, impacted model performance. The performance was only weakly better for models trained on the universal feature set, indicating that bias induced by variable feature size was minimal (**Supplementary Fig. 5f**).

**SUPPLEMENTARY DISCUSSION**

**Study limitations**

We note that our work has several limitations. We used a minimal promoter that provides minimal background expression but could limit the ability to detect potential repressive sequences. We did not confirm that transcripts originated from within the minimal promoter, and results might be different if another common promoter had been used. Conclusions about the properties of enhancers are also caveated by the fact that in MPRAs the distance between enhancer and promoter are short relative to the endogenous genome. Due to oligo synthesis restrictions, we only tested sequence lengths of 200 bp, centered on DNase hypersensitivity peaks, which could overlook additional sequences that are important for cCRE activity. For example, significant MPRA differences were observed when the same genomic region was tested at different lengths^14^. However, it is worth mentioning that DNA accessibility was shown to provide a strong prediction of activity in previous HepG2 and K562 MPRAs that used different tiles that overlap DNase peaks^18^. The sensitivity of DNase peak calling algorithms, which are subjected to sequencing depth and technical bias, could also affect our sequence selection. LentiMPRA tests sequences outside of their genomic context, which could lead to false positives. In addition, the transgene integrates in numerous different genomic locations which could lead to site-of-integration effects. However, to correct for this, we required at least 10 integrations per sequence, had antirepressors on either side of our integrated lentiMPRA construct, and observed high correlations between technical replicates. We also tested sequences in three different cell lines originating from three different donors, which could lead to *trans* environmental effects. However, we should note that previous lentiMPRAs carried out by our lab in different donors for the same cell or organoid type^11^ or even in cell lines from different species^67^ show minor *trans* environmental effects.

**SUPPLEMENTARY METHODS**

**Classical motif enrichment analysis**

*Constructing a non-redundant representative motif subset***.** To reduce redundancy in motif enrichment analysis, we used a non-redundant subset of HOCOMOCO v12 motifs, a derivative of the HOCOMOCO v12 CORE collection^30^. The non-redundant set was constructed as described herein. First, we estimated the motif similarities with MACRO-APE (Vorontsov et al. 2013) at the motif P-value cutoff of 0.0005 and default matrix discretization of 1 (upscaled to 10 to reach a better precision for the cases when similarity estimates with the default discretization exceeded 0.01). Next, using the pairwise motif similarity matrix, we performed hierarchical clustering using sklearn agglomerative clustering ('average' linkage). The number of clusters was taken to maximize the silhouette score, resulting in 523 clusters at the silhouette score of 0.16. Finally, for each cluster, a single representative motif was taken according to the highest average similarity to all other motifs in the cluster (**Supplementary Table 5**). The motif clusters and a non-redundant set of representative motifs are available for downloads from the HOCOMOCO website (https://hocomoco12.autosome.org/downloads_v12).

*Motif enrichment analysis.* We utilized a set of the top 1,000 (i.e., high activity) and bottom 1,000 (i.e., low activity) promoter and potential enhancer sequences for each cell type, as ranked by their MPRA activity. We scanned for motifs by querying these high and low-activity sequences against our aforementioned set of representative motifs using FIMO 5.5.4 (default parameters)^65^ from the MEME-suite. Our search involved using a 0th-order background model created using the input sequences to account for the distribution of individual nucleotides in the sequences. We conducted an enrichment analysis by comparing the number of sequences with motif hits in the high activity set against the number of sequences with motif hits in the low activity set from FIMO results. This allowed us to build a 2x2 contingency table (i.e., the sum of whose entries was 2,000, because we considered a sequence a hit as TRUE if it contained >=1 motif hit) for each motif in each cell type and computed a corresponding Odds Ratio (OR) and p-value using Fisher’s exact test. To visualize the OR for motifs enriched in the low activity class as easily as those in the high activity class, we plotted the log2(OR). We adjusted all p-values for multiple testing using the Benjamini-Hochberg (BH) procedure from the statsmodels library, setting a significance threshold at q-value < 0.0001, or -log10(q-value) > 4.

**Regression modeling**

*Training MPRAnn*. To derive sequence-based features we trained a simple convolutional neural network (CNN), implemented in Tensorflow v2.6.2, with a total of 4 convolutional and 3 dense layers on the large-scale libraries. The complete model architecture is provided (**Supplementary Fig. 7a**). As input, we used the 230bp sequences including the adapters, one-hot encoded them and fit the mean log_2_(RNA/DNA) values from forward and reverse stands. We augmented the batches using the reverse complement of the 200 bp target sequence while keeping the two 15bp adapters fixed. To fit the model, we used a learning rate of 0.001, an early stopping criterion with patience of 10 on 100 epochs, and the Adam optimizer with a mean square error loss function. To make the results directly comparable to the lasso regression model, we trained MPRAnn using 10-fold cross-validation on the identical 10 folds of data as before. For each of the 10 folds, 9 models were trained using each of the remaining folds as a validation set. The final prediction was made on the held-out test set by averaging the predictions across the nine models. For training the joint library, we generated predictions for each of the cell types simultaneously using a multi-task framework. Here, no augmentation was performed because only forward sequences were tested in the MPRA. A snakemake pipeline implementing model training and prediction is available at <https://github.com/visze/sequence_cnn_models>.

**MPRAnn hyperparameter optimization**

The architecture and hyperparameters of MPRAnn were optimized using the Tree of Parzen Estimators (TPE) approach using hyperopt^29,68^, training models on peak centers of DNase-seq data of six frequently studied cell lines in ENCODE (HEK293T, K562, HepG2, HeLa-S3, MCF-7, and GM23338). The input sequence was optimized using sequence lengths of 150, 300, 500, or 1,000 bp. For the convolutional layers, we optimized the number of layers (1-4), the kernel size (6 to 10 for the first two layers, 2 to 4 for the second two layers), the activation function (sigmoid, relu, or softmax), and the number of filters (750, 500, 300, 250, 200, 150, 100, 75, or 50). The stride was always set to one. After the second and fourth convolutional layers, we evaluated whether including a maxpooling layer (pooling: 1 to 3, stride: no stride, 1, or 2) and dropout of 0.1, 0.2, or 0.3 may improve performance. For the two dense layers, we optimized the activation function (sigmoid, softmax) and the number of outputs (100, 200, 300, 400, or 500). Additionally, we studied whether dropout increases the performance after the first dense layer (dropout value: 0.1, 0.2, 0.3, 0.4, or 0.5). In total, we ran 400 training iterations, maximizing the accuracy on the validation set (all peaks on chromosome 18). For each iteration, we used early stopping with a patience of 5, allowing a minimum accuracy delta of 0.001 to judge the results as equal. We ran hyperopt on each cell line individually using a GC-matched background sequence set as negative label as well as on a multitask model with all cell lines included. MPRAnn architecture and hyperparameters were derived from the results of the best models. Results of the best models are shown in **Supplementary Table 7**.

**MPRALegNet architecture and data augmentation strategy**

MPRALegNet used the LegNet architecture^35^, but with the following modifications to properly handle longer sequences and adapt the model to the use of smaller datasets:

i) The initial architecture was designed to solve a soft classification problem that reflects the promoter activity measurements obtained by the fluorescence-based cell sorting into multiple bins. For lentiMPRAs, we replaced the neural network head block with a two-layer MLP [256, 1] to output the continuous expression estimates directly.

ii) To account for longer sequences but smaller training set size compared to the original "yeast MPRA" LegNet, we added the max pooling layers (with kernel size=2 and stride=2) after each local block in order to increase the neural network receptive field while maintaining the same number of parameters.

iii) The kernel size of convolutional blocks and the number of blocks were selected to match the model’s receptive field to the sequence length and to reduce the number of parameters in comparison to the original LegNet. Specifically, for all datasets we used block sizes of [80, 96, 112, 128] for the main part of the network, followed by a mapper block (as in the original paper) consisting of pointwise convolution mapping 128 channels to 256. Using global average pooling, we retrieved 256 values ​​for each sequence, which were then fed into the head block. For HepG2 and K562 datasets we used kernel size ks=11 for the first convolutional layer and 9 for the other convolutions. For WTC11, as this dataset was relatively smaller, we reduced the kernel sizes to 5 and 3, respectively. We verified if the reduced kernel side was beneficial to the model performance using the 1st-to-8th folds for training, and the 9th fold as the validation to avoid any information leakage. This could be possible in the case of directly selecting these parameters using the complete nested 10-fold cross-validation, as in the primary analysis. Of note, reduced kernel size also reduced the neural network receptive field, but still allowed improved performance for the WTC11 data. In the same fashion, we checked the effect of block sizes on the model performance but failed to detect any notable effects. We did not consider increasing the number of blocks for the model as its receptive field was already sufficient for the HepG2 and K562 datasets, and for WTC11 the dataset size was too small to expect the model to learn long-range interactions.

iv) The weight decay during training was increased from 0.01 (the original LegNet) to 0.1 to prevent model overfitting on the smaller training datasets. This particular value was selected from a set of [0.01, 0.05, 0.1, 0.5] using the same training/validation setup with 1-8/9 folds as described above.

v) Gradient clipping (clip_val=1) was used to avoid gradient explosion during the one-cycle learning rate policy.

Of note, we did not perform an exhaustive hyperparameter scan to avoid overfitting the model performance, but we considered several combinations of augmentation strategies, which were compared using 10-fold cross-validation. These augmentations and related model modifications were the following:

(1) Shift augmentation: during training, a sequence could be randomly shifted by 0 to +21 bp.

(2) RevComp augmentation: during training, a supplied sequence could be randomly changed to the reverse complementary orientation.

(3) Orientation augmentation: during training, measured element activity scores were considered separately for each orientation tested, instead of the mean across both orientations used by default.

(4) 5th channel: an extra 5th channel (on top of 4 channels of the one-hot encoding, i.e., the first 4 channels) to explicitly indicate the supplied sequence’s orientation.

(5) Test-time augmentation, whereby the mean prediction was made for various augmentations of the test sequence.

Upon checking multiple possible combinations (**Extended Data Fig. 5a**), the final model for the downstream analysis used all listed augmentations except for the 5th channel augmentation.

**Variant effect prediction**

*Prediction of GWAS-associated SNP effects.* All blood-associated GWAS SNPs in the vicinity of seven disease loci (*GATA1*, *MYC*, *HBE1*, *LMO2*, *RBM38*, *HBA2*, and *BCL11A*) were downloaded as a table from the GWAS catalog^69^. Using a non-redundant list of these lead SNPs filtered to remove indels, we use LDproxy to compute all SNPs in LD (R^2^ ≥ 0.8) with these lead SNPs using all populations encompassed in the 1000 Genomes dataset^70^. For each SNP, we computed a Pred_Ref_-Pred_Alt_ value using our K562 MPRALegNet model to reflect the predicted change in element activity. These values were converted into bedgraph files and visualized on the UCSC genome browser^71^.

*In-silico mutagenesis (ISM) scores on saturation mutagenesis MPRA elements.* All variant effects of *F9*, *LDLR*, *PKLR*, and *SORT1* elements of the saturation mutagenesis MPRA^45^ were downloaded (<https://kircherlab.bihealth.org/satMutMPRA>) because they matched one of the three cell types (HepG2, K562, and WTC11) tested. Next, 1bp deletions were removed from these datasets. ISM scores for all elements were then generated with MPRALegNet using GRCh38 coordinates. Because most of the elements are longer than 200bp element, the regions were tiled in 200bp windows from the beginning with a step size of 150bp, resulting in an overlap of 50bp between neighboring windows. The predictions were averaged on overlapping windows. The missing 30bp input of MPRALegNet was achieved by adding the 15bp adapter to both sides. Prediction and saturation mutagenesis data was compared on all variants associated with a minimum of 10 barcodes.

*Comparing MPRALegNet predictions with allele-specific SNP effects.* To evaluate MPRALegNet against allelic imbalance at allele-specific events (ASEs)^43^, we performed predictions using previously trained MPRALegNet models in 230 bp windows centered at each SNP, considering reference and alternative variants. Here, we used all pre-trained models gathered at the previous stages to predict variant effects in both orientations, resulting in 180+180 predictions for the reference and the alternative alleles, which were then averaged separately. A Pred_Ref_-Pred_Alt_ value was compared against known allelic imbalance. This procedure was performed six times, for ASEs in K562/HepG2 cells and detected in ChIP-Seq/ATAC-Seq/DNase-Seq data as passing 5% FDR in the respective cell types in UDACHA [https://udacha.autosome.org, Release IceKing] and ADASTRA [https://adastra.autosome.org, Release BillCipher]^72^.

*Accounting for prediction uncertainty.* Allele-specific rSNPs are not necessarily linked to gene expression changes. In many cases, the model predictions for alternating alleles are expected to be very similar, thus making it impossible to reliably assess the direction of the predicted effect. To account for the uncertainty in predicting Pred_Ref_-Pred_Alt_, we estimated the standard deviation using the raw scores from all models in the ensemble: *sd* = *sqrt(var_Ref_/N + var_Alt_/N)* where var_Ref_ and var_Alt_ are the prediction variance for Ref and Alt alleles estimated across 90 individual ensemble models providing 180 predictions (considering both orientations). In the case of neutral set variants, the final score distribution would follow a normal distribution with mean = 0 and sd, so we calculated the confidence P-values from z-scores, and used p < 0.05 to select high-confidence predictions.

**REFERENCES**

67. Whalen, S. *et al.* Machine learning dissection of human accelerated regions in primate neurodevelopment. *Neuron* **111**, 857-873.e8 (2023).

68. Bergstra, J., Yamins, D. & Cox, D. Making a science of model search: Hyperparameter optimization in hundreds of dimensions for vision architectures. in *Proceedings of the 30th International Conference on Machine Learning* (eds. Dasgupta, S. & McAllester, D.) vol. 28 115–123 (PMLR, Atlanta, Georgia, USA, 17--19 Jun 2013).

69. Sollis, E. *et al.* The NHGRI-EBI GWAS Catalog: knowledgebase and deposition resource. *Nucleic Acids Res.* **51**, D977–D985 (2023).

70. Auton, A. *et al.* A global reference for human genetic variation. *Nature* **526**, 68–74 (2015).

71. Nassar, L. R. *et al.* The UCSC Genome Browser database: 2023 update. *Nucleic Acids Res.* **51**, D1188–D1195 (2023).

72. Boytsov, A. *et al.* ANANASTRA: annotation and enrichment analysis of allele-specific transcription factor binding at SNPs. *Nucleic Acids Res.* **50**, W51–W56 (2022).
