# Supplementary material for: Massively parallel characterization of transcriptional regulatory elements
Source: Nature. 2025 Jan 15;639(8054):411–20. doi: 10.1038/s41586-024-08430-9 (PMC11903340; doi:10.1038/s41586-024-08430-9)
Supplement: Supplementary file 2 — Reporting Summary [file 41586_2024_8430_MOESM2_ESM.pdf]

nature portfolio

Corresponding author(s): Vikram Agarwal, Jay Shendure, Nadav Ahituv

Last updated by author(s): Oct 13, 2024

Reporting Summary

Nature Portfolio wishes to improve the reproducibility of the work that we publish. This form provides structure for consistency and transparency in reporting. For further information on Nature Portfolio policies, see our Editorial Policies and the Editorial Policy Checklist.

Statistics

For all statistical analyses, confirm that the following items are present in the figure legend, table legend, main text, or Methods section.

n/a	Confirmed	
☐	☒	The exact sample size (*n*) for each experimental group/condition, given as a discrete number and unit of measurement
☐	☒	A statement on whether measurements were taken from distinct samples or whether the same sample was measured repeatedly
☐	☒	The statistical test(s) used AND whether they are one- or two-sided<br>*Only common tests should be described solely by name; describe more complex techniques in the Methods section.*
☐	☒	A description of all covariates tested
☐	☒	A description of any assumptions or corrections, such as tests of normality and adjustment for multiple comparisons
☐	☒	A full description of the statistical parameters including central tendency (e.g. means) or other basic estimates (e.g. regression coefficient) AND variation (e.g. standard deviation) or associated estimates of uncertainty (e.g. confidence intervals)
☐	☒	For null hypothesis testing, the test statistic (e.g. *F*, *t*, *r*) with confidence intervals, effect sizes, degrees of freedom and *P* value noted<br>*Give P values as exact values whenever suitable.*
☒	☐	For Bayesian analysis, information on the choice of priors and Markov chain Monte Carlo settings
☒	☐	For hierarchical and complex designs, identification of the appropriate level for tests and full reporting of outcomes
☐	☒	Estimates of effect sizes (e.g. Cohen's *d*, Pearson's *r*), indicating how they were calculated

Our web collection on statistics for biologists contains articles on many of the points above.

Software and code

Policy information about availability of computer code

Data collection	https://github.com/Altius/hotspot2, https://github.com/ENCODE-DCC/atac-seq-pipeline
Data analysis	bcl2fastq v2.20, MPRAflow 1.0, Enformer v1.0, https://github.com/visze/sequence_cnn_models, https://github.com/autosome-ru/human_legnet, TF-MoDISco-lite v2.0.4, Tomtom, STREME, https://github.com/jmschrei/apricot/

For manuscripts utilizing custom algorithms or software that are central to the research but not yet described in published literature, software must be made available to editors and reviewers. We strongly encourage code deposition in a community repository (e.g. GitHub). See the Nature Portfolio guidelines for submitting code & software for further information.

Data

Policy information about availability of data

All manuscripts must include a data availability statement. This statement should provide the following information, where applicable:

- Accession codes, unique identifiers, or web links for publicly available datasets
- A description of any restrictions on data availability
- For clinical datasets or third party data, please ensure that the statement adheres to our policy

Raw sequencing data and processed files generated in this study are available in the ENCODE portal for the pilot libraries (HepG2: ENCSR463IRX; K562: ENCSR460LZI), large-scale libraries (HepG2: ENCSR022GQD; K562: ENCSR382BVV; WTC11: ENCSR244FWB), and joint libraries (HepG2: ENCSR405QCT; K562: ENCSR203UFY; WTC11: ENCSR336MKI). Code to train and interpret MPRAnn and MPRALegNet is available at https://github.com/visze/sequence_cnn_models and https://github.com/autosome-ru/human_legnet. Pretrained models and code have also been deposited in Zenodo (https://zenodo.org/record/8219231). All

summarized processed data is included in supplementary tables.

Human research participants

Policy information about studies involving human research participants and Sex and Gender in Research.

Reporting on sex and gender	We did not perform sex- and gender-based analysis in the study, because the data were collected using established cell lines. Gender has no impact on the lentiMPRA results.
Population characteristics	Population characteristics are not relevant to this study.
Recruitment	Recruitment is not relevant to this study.
Ethics oversight	N/A

Note that full information on the approval of the study protocol must also be provided in the manuscript.

Field-specific reporting

Please select the one below that is the best fit for your research. If you are not sure, read the appropriate sections before making your selection.

[x] Life sciences [ ] Behavioural & social sciences [ ] Ecological, evolutionary & environmental sciences

For a reference copy of the document with all sections, see nature.com/documents/nr-reporting-summary-flat.pdf

Life sciences study design

All studies must disclose on these points even when the disclosure is negative.

Sample size	Sample sizes are noted in each of the figures and resulted from the number of elements designed that were successfully measured after using our processing pipeline.
Data exclusions	Low quality data excluded from the analysis are clearly indicated in the Methods sections with described filtering procedures
Replication	LentiMPRA were done with three biological replicates for each of three cell types (HepG2, K562, WTC11).
Randomization	N/A
Blinding	Blinding was not possible, as we needed to know the identity of each library for the subsequent analyses.

Reporting for specific materials, systems and methods

We require information from authors about some types of materials, experimental systems and methods used in many studies. Here, indicate whether each material, system or method listed is relevant to your study. If you are not sure if a list item applies to your research, read the appropriate section before selecting a response.

Materials & experimental systems

n/a	Involved in the study	
[x]	[ ]	Antibodies
[ ]	[x]	Eukaryotic cell lines
[x]	[ ]	Palaeontology and archaeology
[x]	[ ]	Animals and other organisms
[x]	[ ]	Clinical data
[x]	[ ]	Dual use research of concern

Methods

n/a	Involved in the study	
[x]	[ ]	ChIP-seq
[x]	[ ]	Flow cytometry
[x]	[ ]	MRI-based neuroimaging

Eukaryotic cell lines

Policy information about cell lines and Sex and Gender in Research

Cell line source(s)	293T (CRL-3216, ATCC), HepG2 (HB-8065, ATCC), K562 (CCL-243, ATCC), WTC11 (GM25256, Coriell Institute).
Authentication	293T, HepG2, K562 and WTC11 cells were not authenticated.
Mycoplasma contamination	293T, HepG2, K562 and WTC11 cells were not tested for mycoplasma contamination.
Commonly misidentified lines (See ICLAC register)	No commonly misidentified cell lines were used in the study.
