# Supplementary material for: Massively parallel characterization of transcriptional regulatory elements
Source: Nature. 2025 Jan 15;639(8054):411–20. doi: 10.1038/s41586-024-08430-9 (PMC11903340; doi:10.1038/s41586-024-08430-9)
Supplement: Supplementary file 3 — Supplementary Figs. 1–11 [file 41586_2024_8430_MOESM3_ESM.pdf]

Supplementary Fig. 1

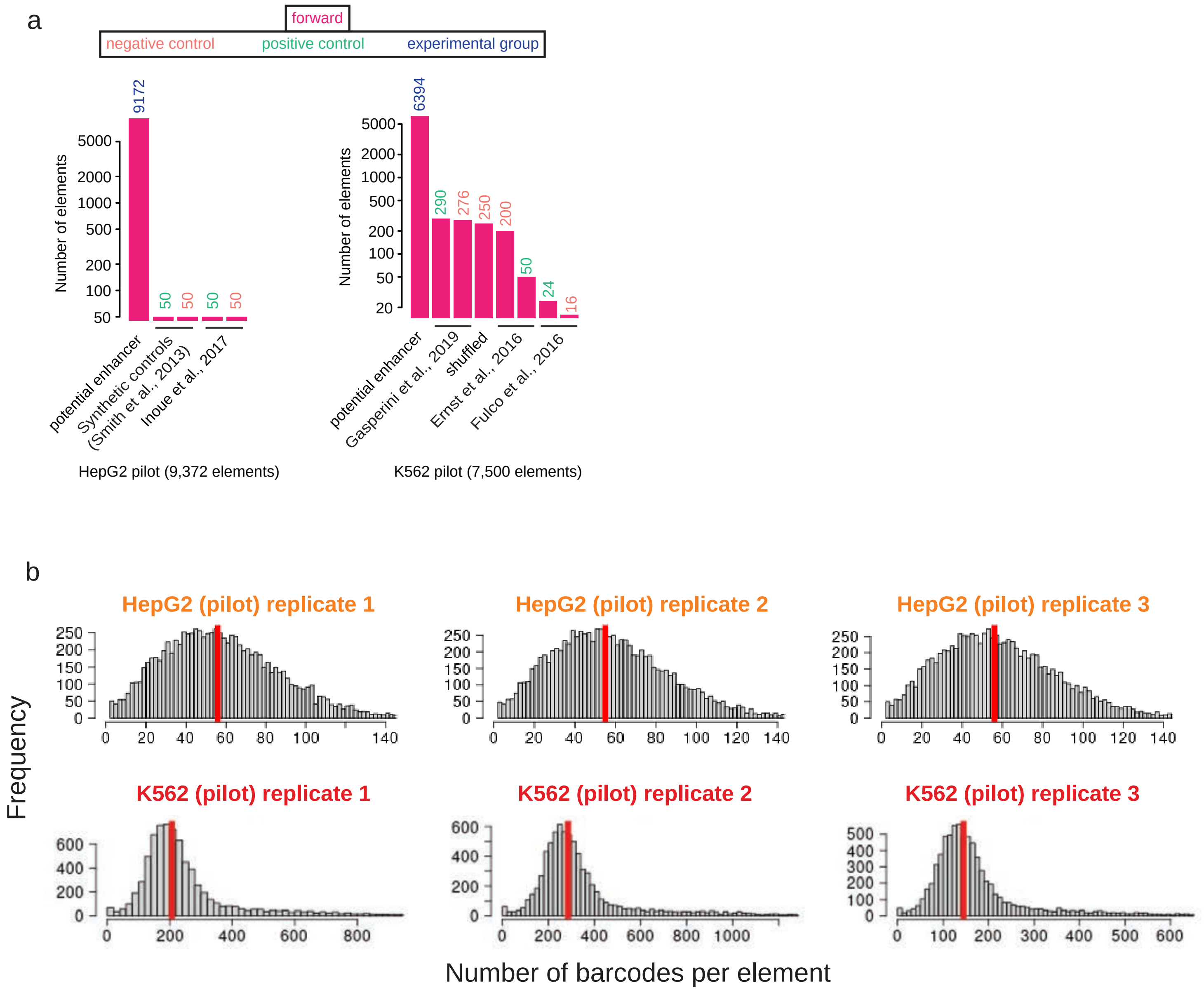

Supplementary Fig. 1

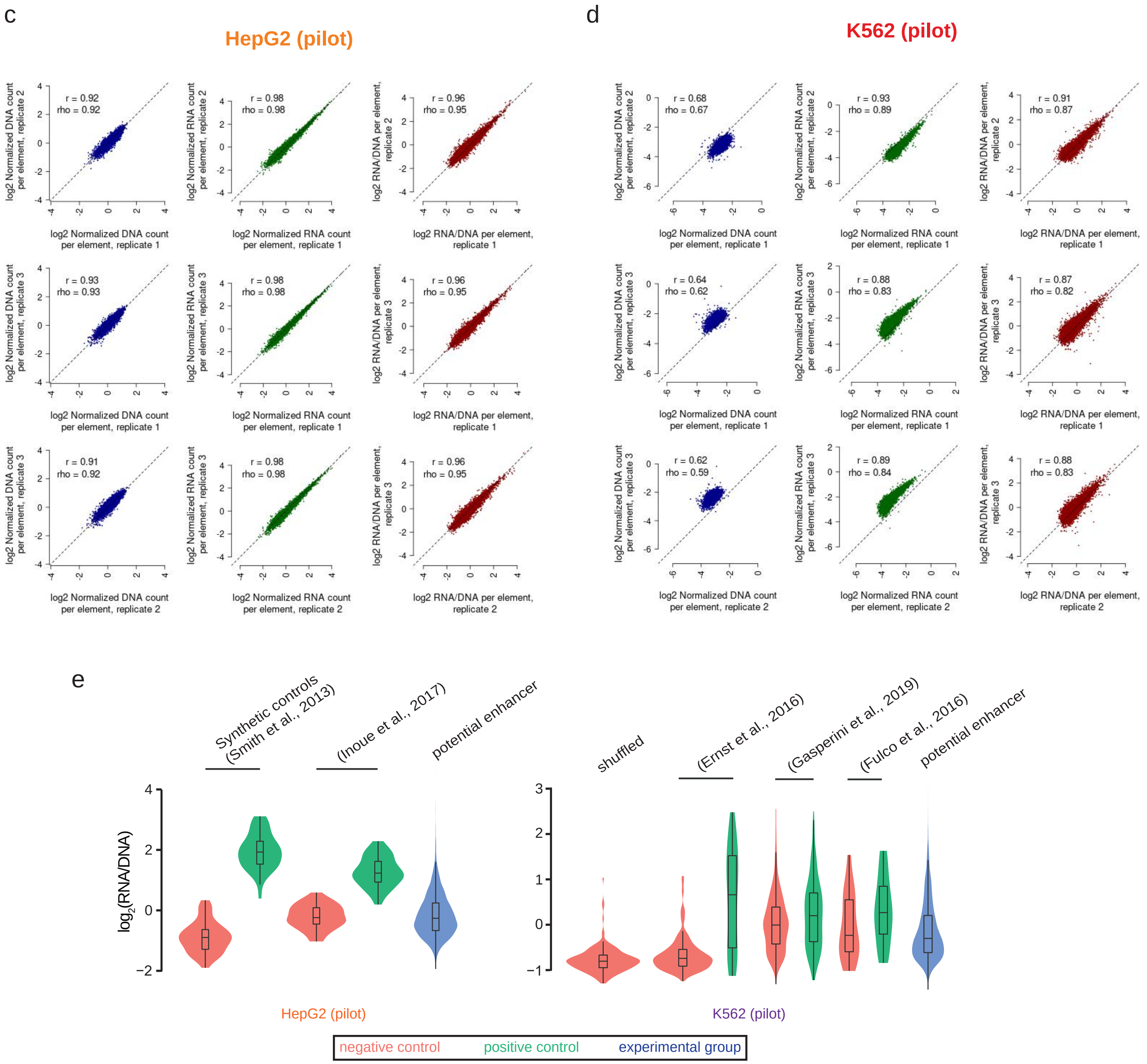

Supplementary Fig. 1: Design and quality control characteristics of two pilot MPRA libraries. a, Composition of the HepG2 and K562 pilot libraries. Thousands of potential enhancers, negative controls (dinucleotide shuffled sequences or elements lacking a signal from prior studies), and positive controls (elements with reported activity from prior studies) are included in each library. To maintain consistency with **Fig. 1b**, bars are colored according to orientation tested, with accompanying numbers indicating the number of elements tested in the category. Numbers are colored according to element type. **b,** Shown are histograms indicating the number of observed barcodes per element, for each of the three replicates and two pilot MPRA libraries. Shown with a vertical red line is the median number of barcodes per element. **c-d,** Shown are scatter plots displaying the relationship between observed DNA counts (blue), RNA counts (green), and RNA/DNA ratios (red) for all pairwise comparisons among replicates, for both the **(c)** HepG2 and **(d)** K562 pilot MPRA libraries. Also indicated is the Pearson (*r*) and Spearman (rho) correlation values. Candidate enhancers supported by fewer than 10 barcodes were filtered out prior to this analysis to reduce the impact of technical noise. **e,** Violin plots of element activity [measured as $\log_2$(RNA/DNA)] for potential enhancers, negative controls, and positive controls for each library.

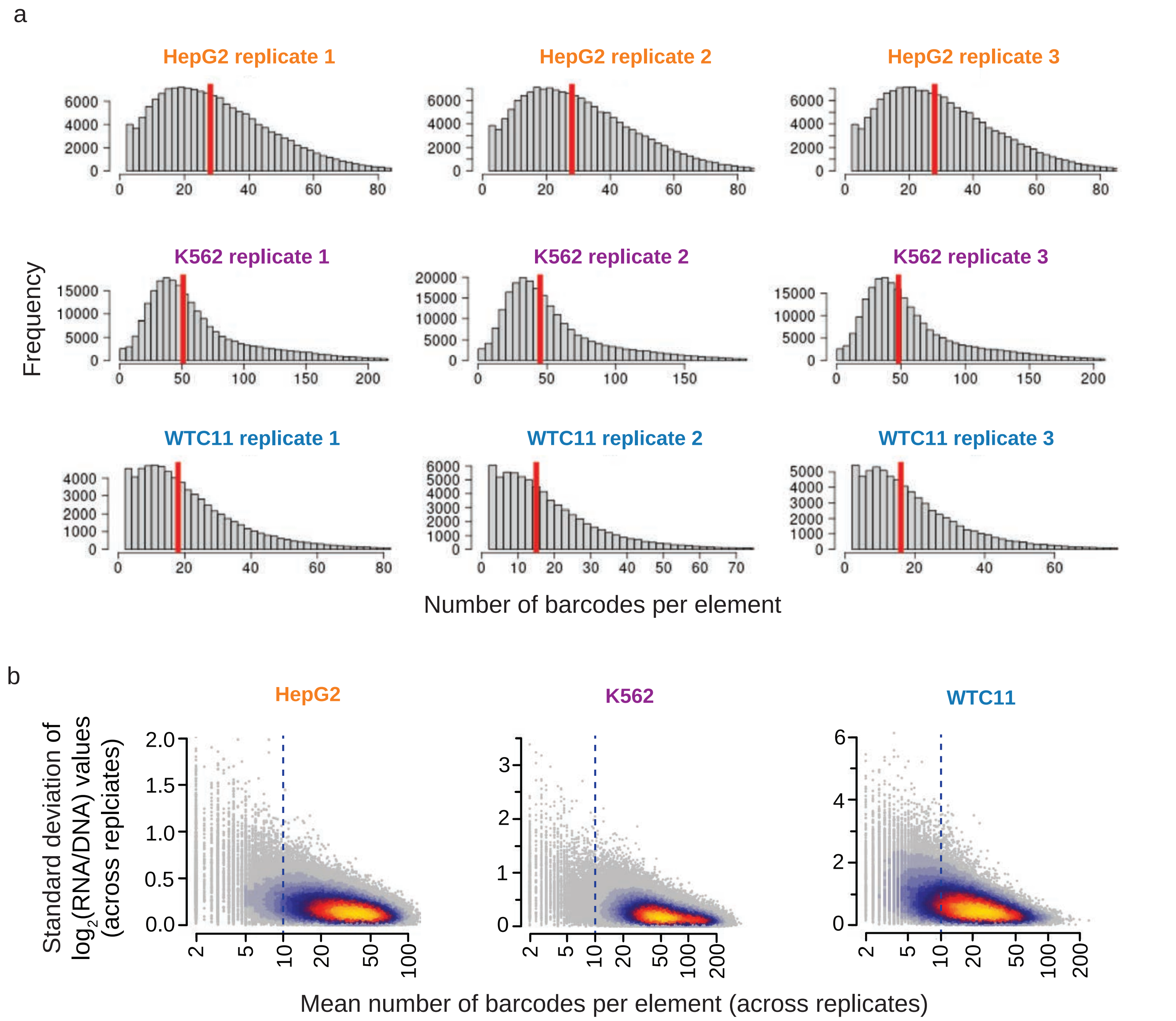
a
HepG2 replicate 1
HepG2 replicate 2
HepG2 replicate 3
K562 replicate 1
K562 replicate 2
K562 replicate 3
WTC11 replicate 1
WTC11 replicate 2
WTC11 replicate 3
Frequency
Number of barcodes per element
b
HepG2
K562
WTC11
Standard deviation of log2(RNA/DNA) values (across replciates)
Mean number of barcodes per element (across replicates)

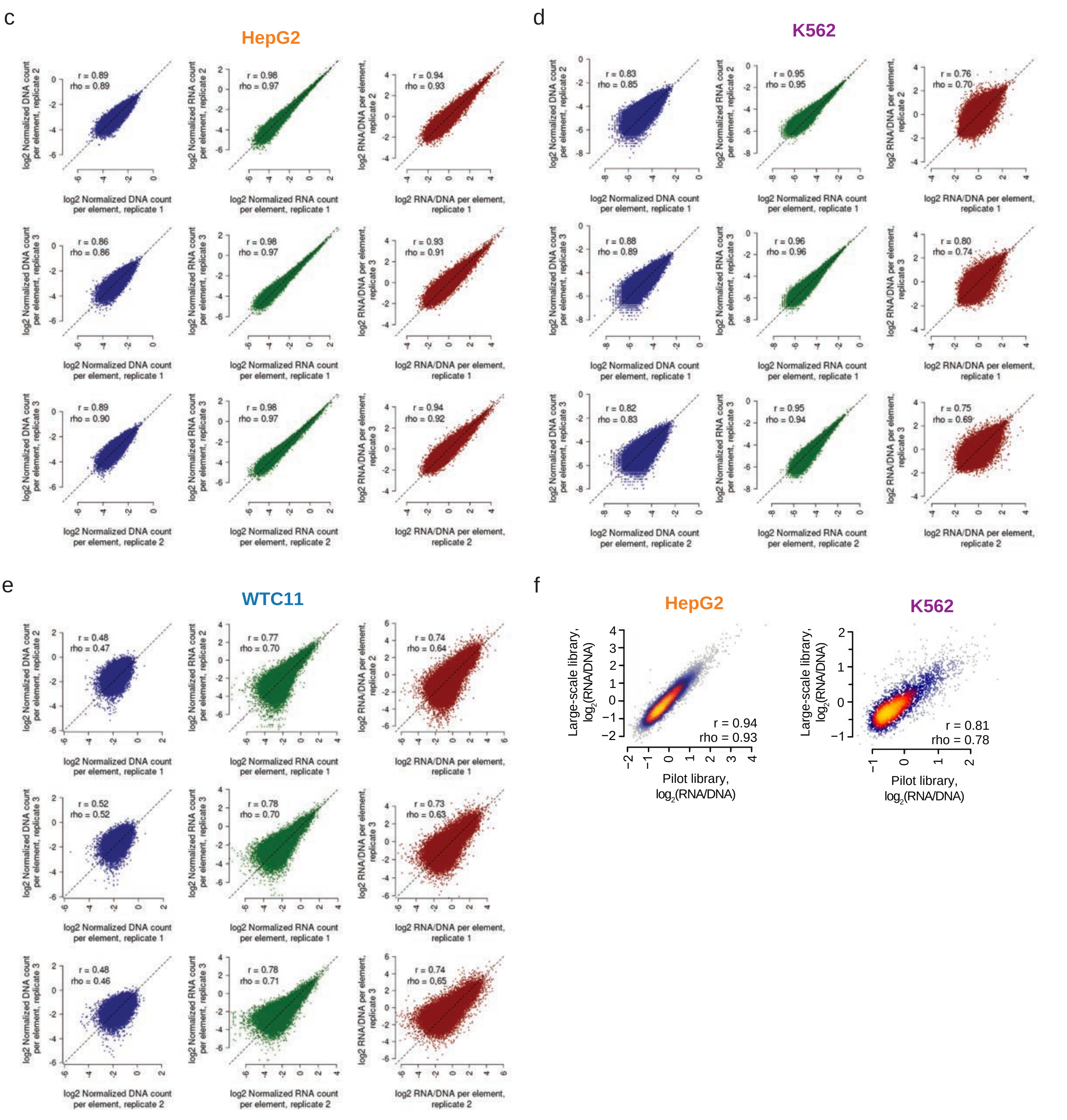
c
HepG2
r = 0.89
rho = 0.89
log2 Normalized DNA count per element, replicate 2
log2 Normalized DNA count per element, replicate 1
r = 0.98
rho = 0.97
log2 Normalized RNA count per element, replicate 2
log2 Normalized RNA count per element, replicate 1
r = 0.94
rho = 0.93
log2 RNA/DNA per element, replicate 2
log2 RNA/DNA per element, replicate 1
r = 0.86
rho = 0.86
log2 Normalized DNA count per element, replicate 3
log2 Normalized DNA count per element, replicate 1
r = 0.98
rho = 0.97
log2 Normalized RNA count per element, replicate 3
log2 Normalized RNA count per element, replicate 1
r = 0.93
rho = 0.91
log2 RNA/DNA per element, replicate 3
log2 RNA/DNA per element, replicate 1
r = 0.89
rho = 0.90
log2 Normalized DNA count per element, replicate 3
log2 Normalized DNA count per element, replicate 2
r = 0.98
rho = 0.97
log2 Normalized RNA count per element, replicate 3
log2 Normalized RNA count per element, replicate 2
r = 0.94
rho = 0.92
log2 RNA/DNA per element, replicate 3
log2 RNA/DNA per element, replicate 2
d
K562
r = 0.83
rho = 0.85
r = 0.95
rho = 0.95
r = 0.76
rho = 0.70
r = 0.88
rho = 0.89
r = 0.96
rho = 0.96
r = 0.80
rho = 0.74
r = 0.82
rho = 0.83
r = 0.95
rho = 0.94
r = 0.75
rho = 0.69
e
WTC11
r = 0.48
rho = 0.47
r = 0.77
rho = 0.70
r = 0.74
rho = 0.64
r = 0.52
rho = 0.52
r = 0.78
rho = 0.70
r = 0.73
rho = 0.63
r = 0.48
rho = 0.46
r = 0.78
rho = 0.71
r = 0.74
rho = 0.65
f
HepG2
Large-scale library, log2(RNA/DNA)
r = 0.94
rho = 0.93
Pilot library, log2(RNA/DNA)
K562
Large-scale library, log2(RNA/DNA)
r = 0.81
rho = 0.78
Pilot library, log2(RNA/DNA)

Supplementary Fig. 2

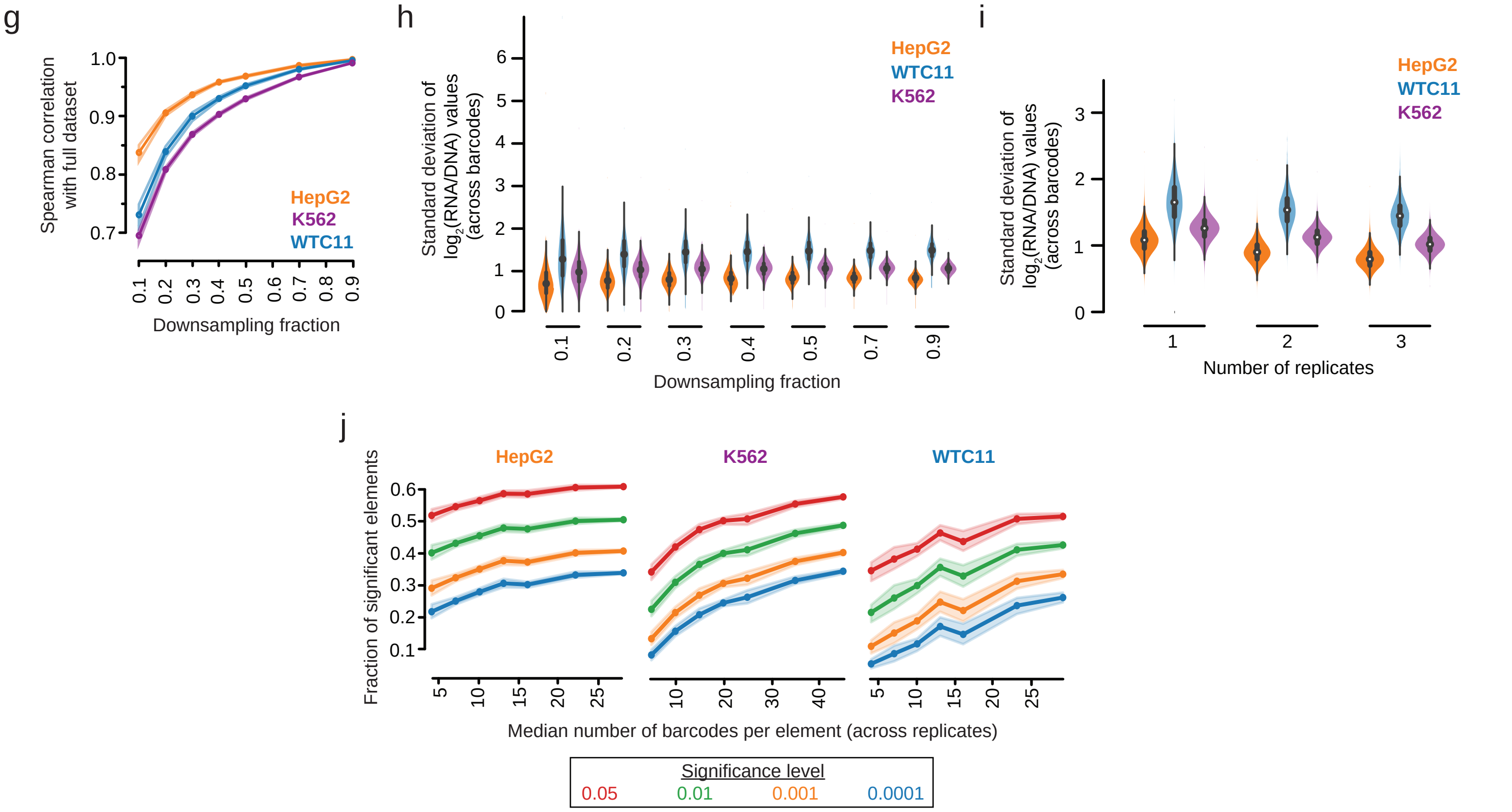

Supplementary Fig. 2: Quality control characteristics of the three large-scale MPRA libraries. a, Shown are histograms indicating the number of observed barcodes per element, for each of the three replicates and three large-scale MPRA libraries. Shown with a vertical red line is the median number of barcodes per element. **b,** Relationship between the mean number of barcodes in each library relative to the standard deviation of the element activity scores across replicates. The dashed line indicates our threshold of 10 minimum barcodes. **c-e,** Shown are scatter plots displaying the relationship between observed DNA counts (blue), RNA counts (green), and RNA/DNA ratios (red) for all pairwise comparisons among replicates, for the **(c)** HepG2, **(d)** K562, and **(e)** WTC11 large-scale MPRA libraries. Candidate elements supported by fewer than 10 barcodes were filtered out prior to this analysis to reduce the impact of technical noise. **f,** Scatter plots displaying the relationships between activity scores for the subset of elements common to both the pilot and large-scale libraries tested in HepG2 and K562 cells. Also indicated is the Pearson (*r*) and Spearman (rho) correlation values. **g,** Correlation between the $\log_2$(RNA/DNA) ratios of the downsampled and the full data. Barcodes were downsampled at fractions of 0.1, 0.2, 0.3, 0.4, 0.5, 0.7, and 0.9 of the total number of barcodes per sequence. The plot shows the average and the 95% confidence interval of 10 downsampling replicates for HepG2, WTC11, and K562 cells. **h,** Standard deviation between barcode $\log_2$(RNA/DNA) ratios associated to the same element at downsampling fractions of 0.1, 0.2, 0.3, 0.4, 0.5, 0.7, and 0.9 for HepG2, WTC11, and K562. For each sequence and downsampling fraction the downsampling was performed 10 times. **i,** Standard deviation between barcode $\log_2$(RNA/DNA) ratios associated to the same element when using one, two, or three replicates for HepG2, WTC11, and K562 cells. **j,** Fraction of all sequences in HepG2, WTC11, and K562 cells that are found to be significantly active compared to the shuffled negative controls after downsampling barcodes at downsampling fractions of 0.1, 0.2, 0.3, 0.4, 0.5, 0.7, and 0.9. On the x-axis, the median number of barcodes after downsampling is displayed. The y-axis shows the average fraction and the 95% confidence interval of 10 downsampling replicates at four different significance levels. The significance of each sequence was calculated using the empirical p-value based on the MAD-score from the distribution of the downsampled shuffled negative controls.

Supplementary Fig. 3

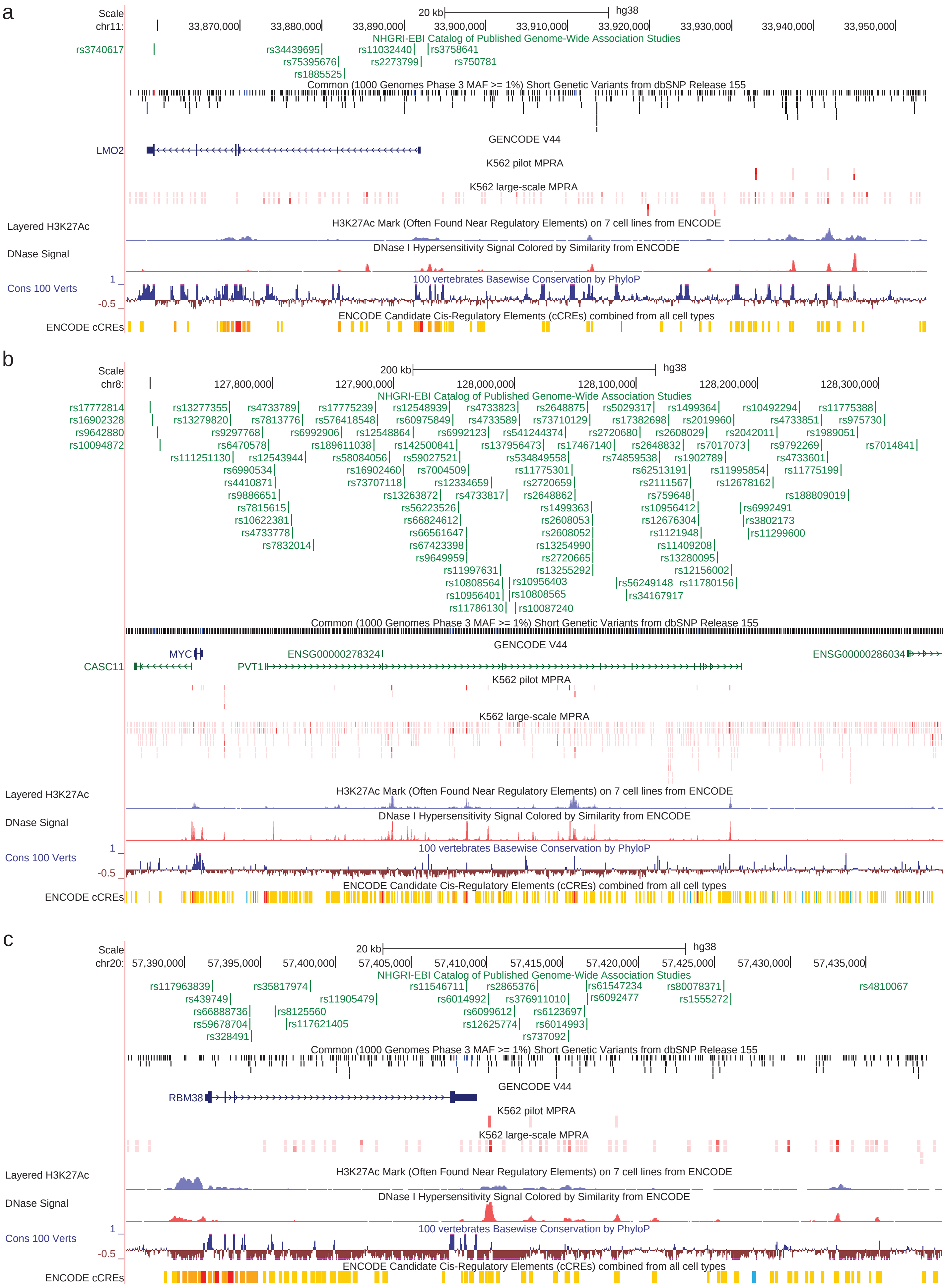

Supplementary Fig. 3: MPRA activity in selected disease loci. a-c, This figure is an extension of **Extended Data Fig. 2** but displays *LMO2* (**a**), *MYC* (**b**), and *RBM38* (**c**) as additional loci.

a

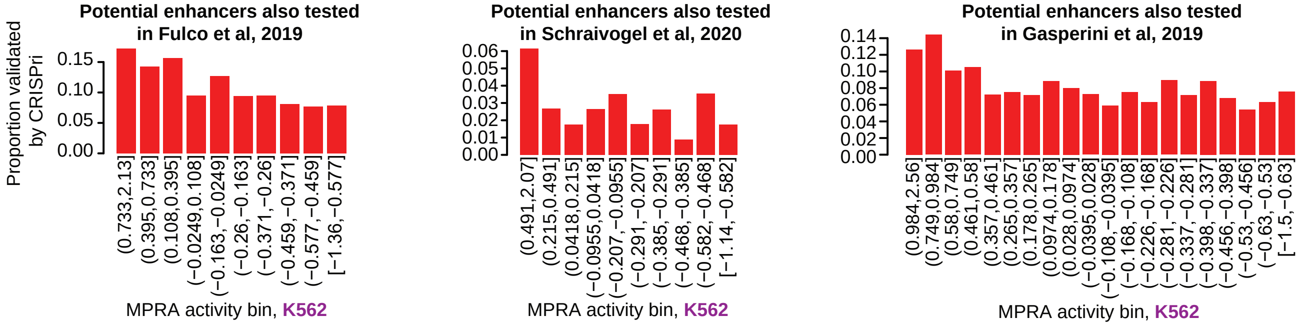

b

Fulco et al, 2019

	Estimate	Std. Error	z value	Pr(>|z|)	
(Intercept)	-3.750821	0.163289	-22.970	< 2e-16	***
ABC	1.679589	0.149541	11.232	< 2e-16	***
MPRA	-0.006809	0.158644	-0.043	0.96577	
ABC:MPRA	-0.303745	0.078973	-3.846	0.00012	***

Schraivogel et al, 2020

	Estimate	Std. Error	z value	Pr(>|z|)	
(Intercept)	-5.44408	0.21690	-25.099	< 2e-16	***
ABC	0.39039	0.05555	7.027	2.11e-12	***
MPRA	0.34648	0.17401	1.991	0.0465	*
ABC:MPRA	-0.06443	0.06779	-0.950	0.3419	

Gasperini et al, 2019

	Estimate	Std. Error	z value	Pr(>|z|)	
(Intercept)	-4.59639	0.19142	-24.012	<2e-16	***
ABC	0.62208	0.07319	8.499	<2e-16	***
MPRA	-0.23394	0.20270	-1.154	0.248	
ABC:MPRA	0.03669	0.07784	0.471	0.637	

Signif. codes: 0 ‘***’ 0.001 ‘**’ 0.01 ‘*’ 0.05

Supplementary Fig. 4: Relationship between MPRA and CRISPRi datasets. a, Fraction of elements validated by CRISPRi (*i.e.*, called to significantly regulate a target gene) for equally sized bins of MPRA activity. The bins with the highest activity level exhibit a greater likelihood for validation. The left and right numerical intervals of the MPRA activity bins are shown comma-separated, with open parentheses meaning the exclusion of the left interval and square brackets meaning the inclusion of the right interval. Three CRISPRi datasets were cross-referenced for this analysis (Fulco et al., 2019, Gasperini et al., 2019, Schraivogel et al., 2020). **b,** Using the datasets in part (a), we intersected our set of potential enhancers with those tested by CRISPRi. We then trained a binary classifier, using logistic regression to predict whether a specific enhancer-gene linkage was statistically significant, considering the ABC model along with an interaction term with MPRA activity. The tables shown indicate the coefficients, z-value, and statistical significance of each term. The Fulco et al., 2019 and Schraivogel et al., 2020 datasets exhibited support for at least one MPRA term while there was no evidence for the benefit of this term in the Gasperini et al., 2019 dataset.

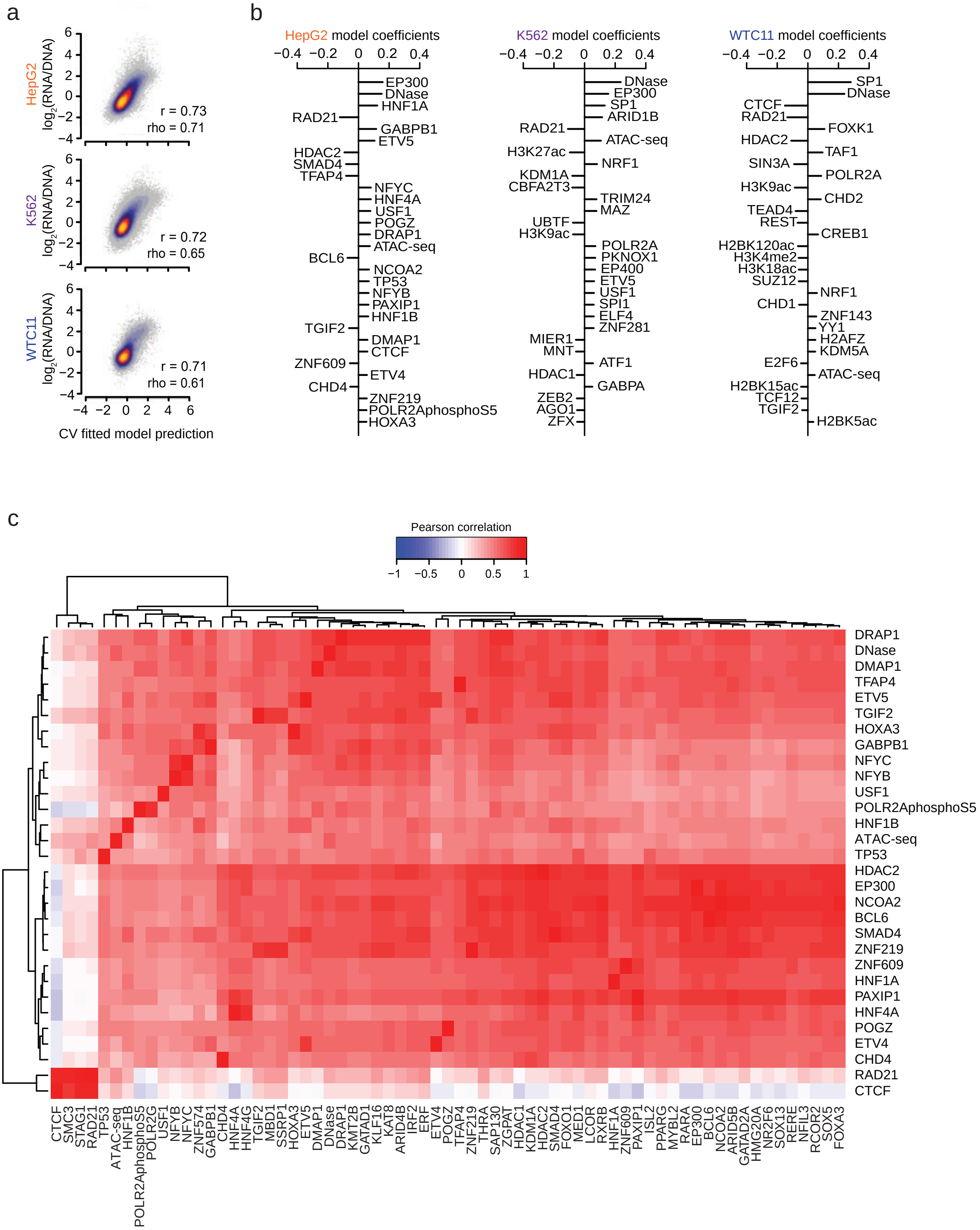
a
HepG2
log2(RNA/DNA)
r = 0.73
rho = 0.71
K562
log2(RNA/DNA)
r = 0.72
rho = 0.65
WTC11
log2(RNA/DNA)
r = 0.71
rho = 0.61
−4 −2 0 2 4 6
CV fitted model prediction
b
HepG2 model coefficients
−0.4 −0.2 0 0.2 0.4
EP300
DNase
HNF1A
RAD21
GABPB1
ETV5
HDAC2
SMAD4
TFAP4
NFYC
HNF4A
USF1
POGZ
DRAP1
ATAC-seq
BCL6
NCOA2
TP53
NFYB
PAXIP1
HNF1B
TGIF2
DMAP1
CTCF
ZNF609
ETV4
CHD4
ZNF219
POLR2AphosphoS5
HOXA3
K562 model coefficients
−0.4 −0.2 0 0.2 0.4
DNase
EP300
SP1
ARID1B
RAD21
ATAC-seq
H3K27ac
NRF1
KDM1A
CBFA2T3
TRIM24
MAZ
UBTF
H3K9ac
POLR2A
PKNOX1
EP400
ETV5
USF1
SPI1
ELF4
ZNF281
MIER1
MNT
ATF1
HDAC1
GABPA
ZEB2
AGO1
ZFX
WTC11 model coefficients
−0.4 −0.2 0 0.2 0.4
SP1
DNase
CTCF
RAD21
FOXK1
HDAC2
TAF1
SIN3A
POLR2A
H3K9ac
CHD2
TEAD4
REST
CREB1
H2BK120ac
H3K4me2
H3K18ac
SUZ12
NRF1
CHD1
ZNF143
YY1
H2AFZ
KDM5A
E2F6
ATAC-seq
H2BK15ac
TCF12
TGIF2
H2BK5ac
c
Pearson correlation
−1 −0.5 0 0.5 1
DRAP1
DNase
DMAP1
TFAP4
ETV5
TGIF2
HOXA3
GABPB1
NFYC
NFYB
USF1
POLR2AphosphoS5
HNF1B
ATAC-seq
TP53
HDAC2
EP300
NCOA2
BCL6
SMAD4
ZNF219
ZNF609
HNF1A
PAXIP1
HNF4A
POGZ
ETV4
CHD4
RAD21
CTCF
CTCF
SMC3
STAG1
RAD21
TP53
ATAC-seq
HNF1B
POLR2AphosphoS5
POLR2G
USF1
NFYB
NFYC
ZNF574
GABPB1
CHD4
HNF4A
HNF4G
TGIF2
MBD1
SSRP1
HOXA3
ETV5
DMAP1
DNase
DRAP1
KMT2B
GATAD1
KLF16
KAT8
ARID4B
IRF2
ERF
ETV4
POGZ
TFAP4
ZNF219
THRA
SAP130
ZGPAT
HDAC1
KDM1A
HDAC2
SMAD4
FOXO1
MED1
LCOR
RXRB
HNF1A
ZNF609
PAXIP1
ISL2
PPARG
MYBL2
RARA
EP300
BCL6
NCOA2
ARID5B
GATAD2A
HMG20A
NR2F6
SOX13
RERE
NFIL3
RCOR2
SOX5
FOXA3

d

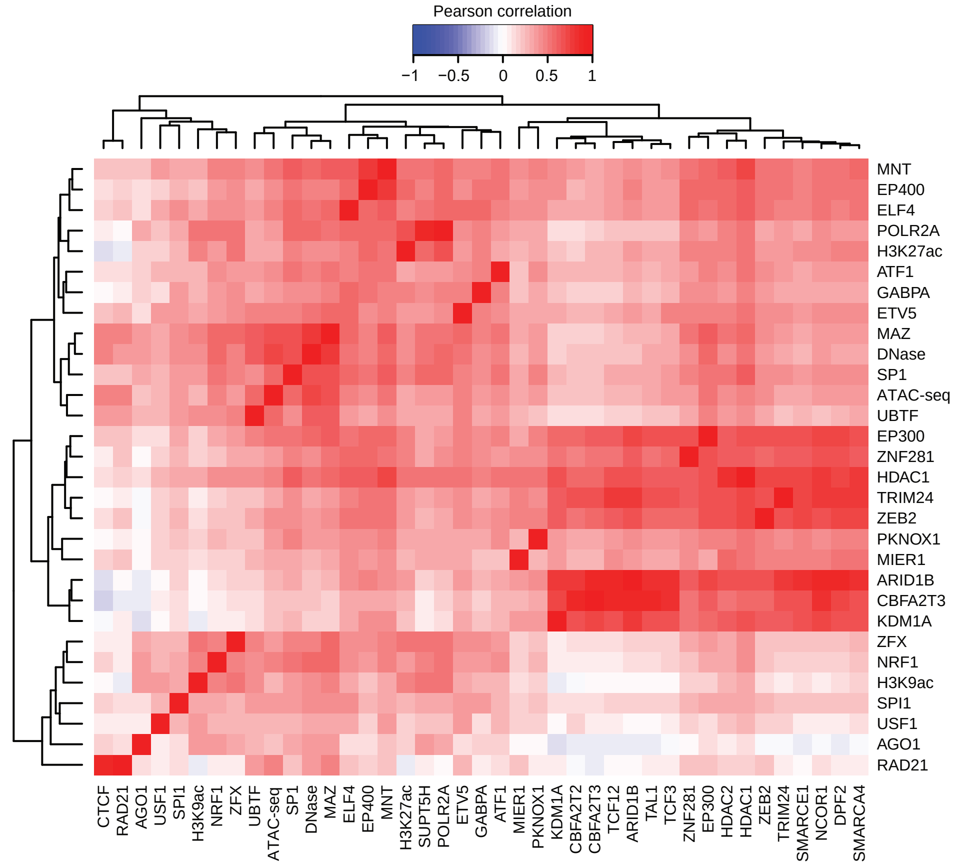
Pearson correlation
−1
−0.5
0
0.5
1
MNT
EP400
ELF4
POLR2A
H3K27ac
ATF1
GABPA
ETV5
MAZ
DNase
SP1
ATAC-seq
UBTF
EP300
ZNF281
HDAC1
TRIM24
ZEB2
PKNOX1
MIER1
ARID1B
CBFA2T3
KDM1A
ZFX
NRF1
H3K9ac
SPI1
USF1
AGO1
RAD21
CTCF
RAD21
AGO1
USF1
SPI1
H3K9ac
NRF1
ZFX
UBTF
ATAC-seq
SP1
DNase
MAZ
ELF4
EP400
MNT
H3K27ac
SUPT5H
POLR2A
ETV5
GABPA
ATF1
MIER1
PKNOX1
KDM1A
CBFA2T2
CBFA2T3
TCF12
ARID1B
TAL1
TCF3
ZNF281
EP300
HDAC2
HDAC1
ZEB2
TRIM24
SMARCE1
NCOR1
DPF2
SMARCA4

e

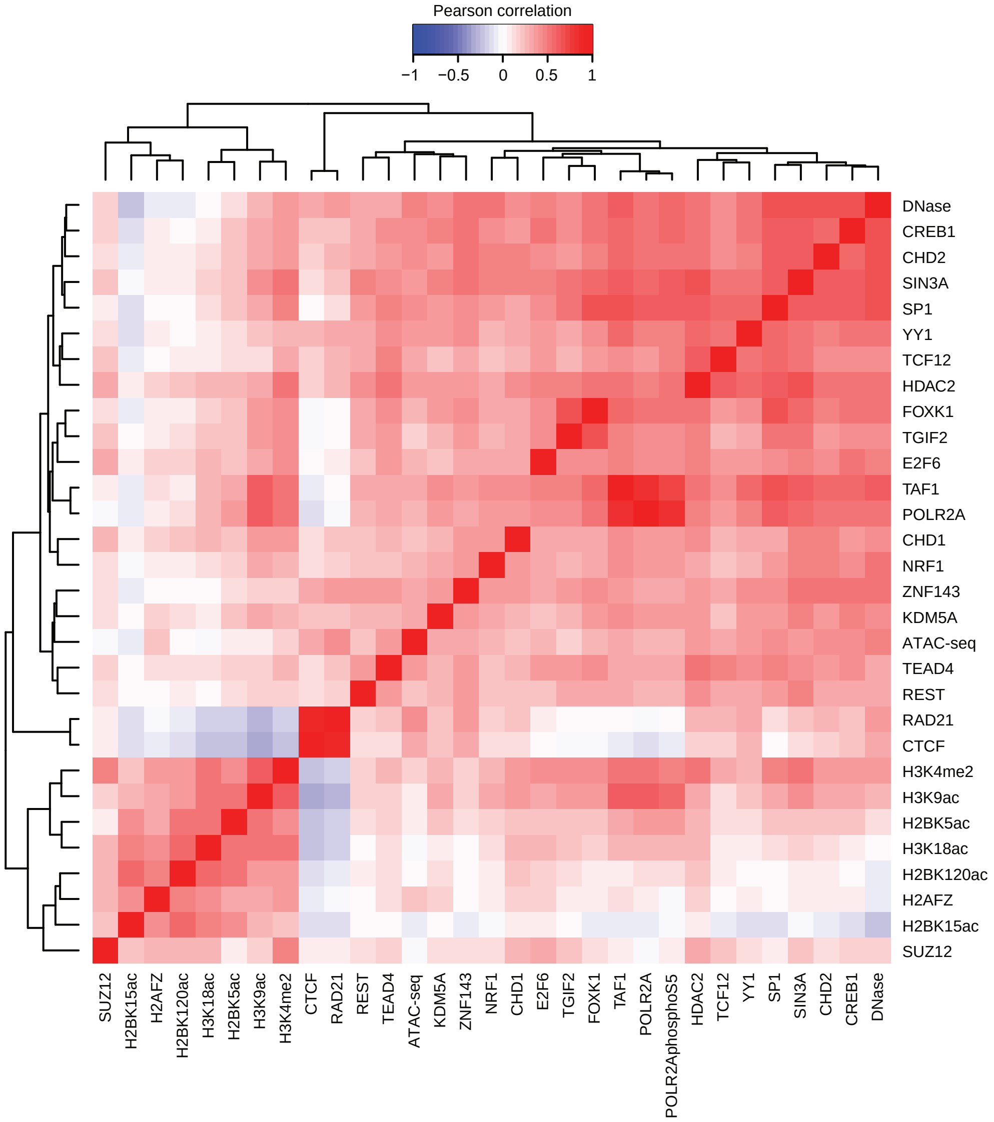
Pearson correlation
−1
−0.5
0
0.5
1
DNase
CREB1
CHD2
SIN3A
SP1
YY1
TCF12
HDAC2
FOXK1
TGIF2
E2F6
TAF1
POLR2A
CHD1
NRF1
ZNF143
KDM5A
ATAC-seq
TEAD4
REST
RAD21
CTCF
H3K4me2
H3K9ac
H2BK5ac
H3K18ac
H2BK120ac
H2AFZ
H2BK15ac
SUZ12
SUZ12
H2BK15ac
H2AFZ
H2BK120ac
H3K18ac
H2BK5ac
H3K9ac
H3K4me2
CTCF
RAD21
REST
TEAD4
ATAC-seq
KDM5A
ZNF143
NRF1
CHD1
E2F6
TGIF2
FOXK1
TAF1
POLR2A
POLR2AphosphoS5
HDAC2
TCF12
YY1
SP1
SIN3A
CHD2
CREB1
DNase

f

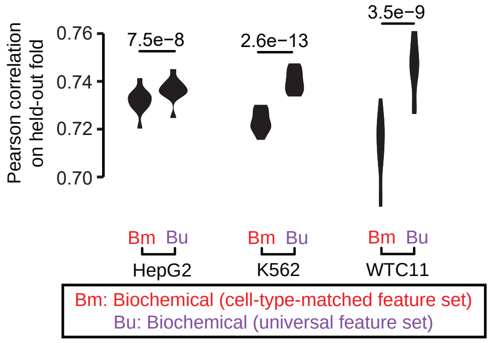

Pearson correlation on held-out fold
0.76
0.74
0.72
0.70
7.5e−8
2.6e−13
3.5e−9
Bm Bu
HepG2
Bm Bu
K562
Bm Bu
WTC11
Bm: Biochemical (cell-type-matched feature set)
Bu: Biochemical (universal feature set)

Supplementary Fig. 5: Prediction of sequence activity based on biochemical features. a, Scatter plot indicating relationship between model predictions and observed element activity scores for each cell type. Pearson (r) and Spearman (rho) correlation values are shown after concatenating the observations for all 10 cross-validation folds of held-out data. **b,** The top 30 coefficients derived from lasso regression models trained independently on each cell type. **c-e,** Pearson correlation matrix between the top 30 features from **panel b**, shown as rows, and other features sharing a Pearson correlation either ≤ -0.8 or ≥ 0.8, shown as columns, for HepG2 (**c**), K562 (**d**), and WTC11 (**e**) cells. Hierarchical clustering was used to group features exhibiting similar correlation patterns. **f,** Violin plots showing the performances of the trained biochemical models on each of the ten cross-validation folds of held-out data. The lasso regression models evaluated are those that consider cell-type matched features (Bm) for the indicated cell type, or those that consider a universal set of biochemical features across all three cell types (Bu). An improvement relative to another model was evaluated with a one-sided, paired t-test.

Supplementary Fig. 6

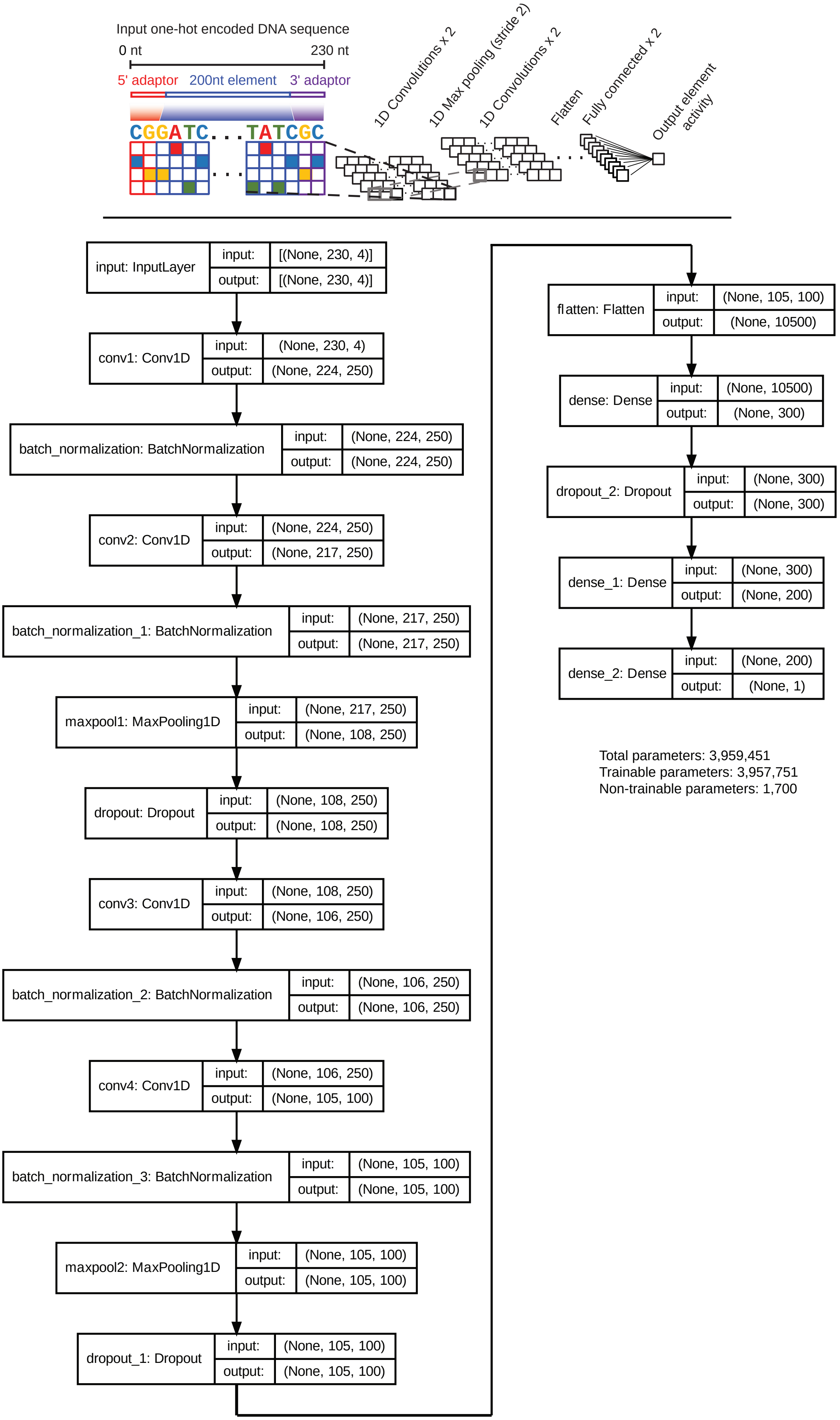

Supplementary Fig. 6: Architecture of MPRAnn. Cartoon (top) and complete architecture (bottom) of the MPRAnn model. Indicated for each layer is the layer name and dimensionality of the input and output matrices. 'None' refers to the batch size used during model training.

Supplementary Fig. 7

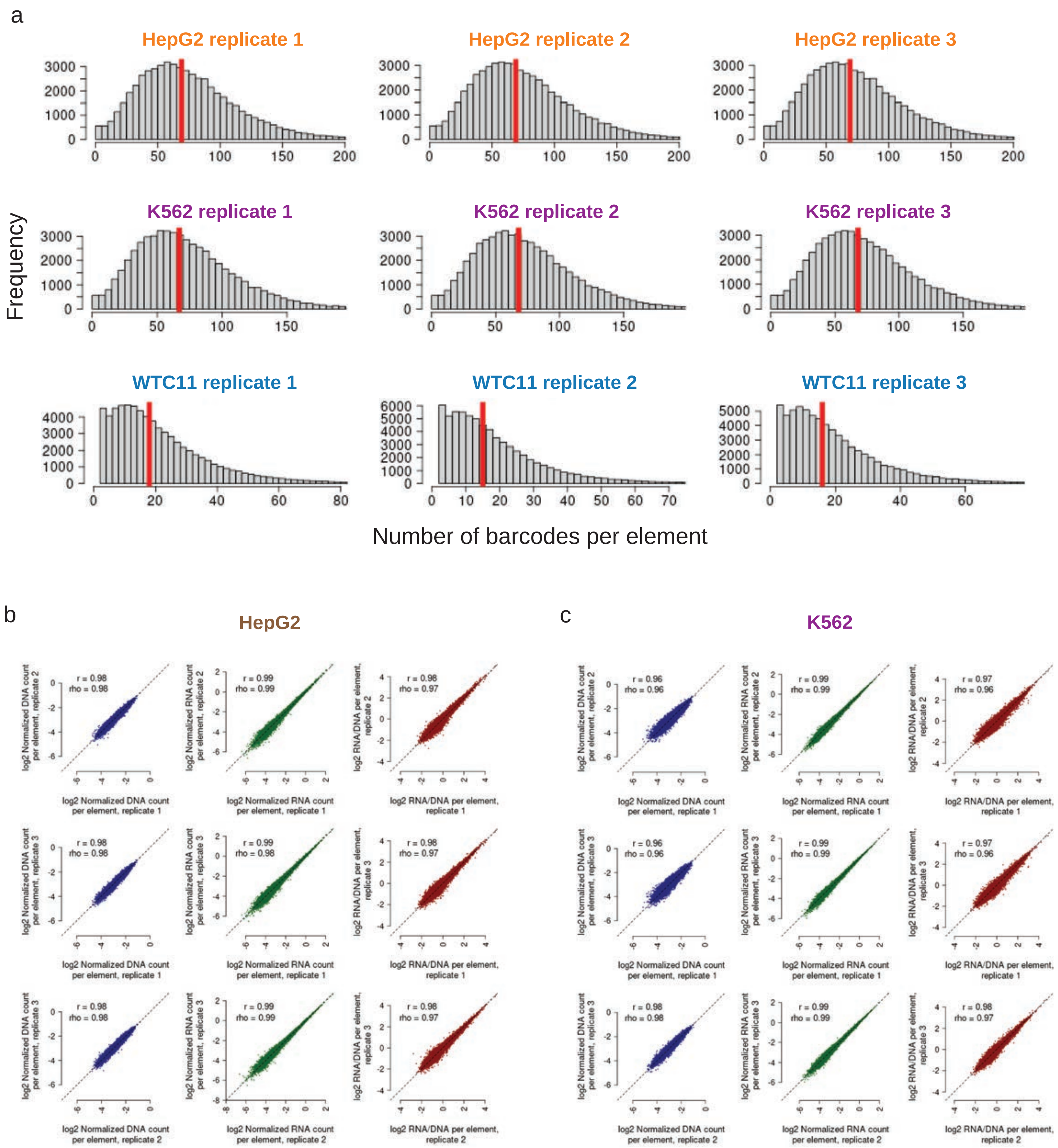

d

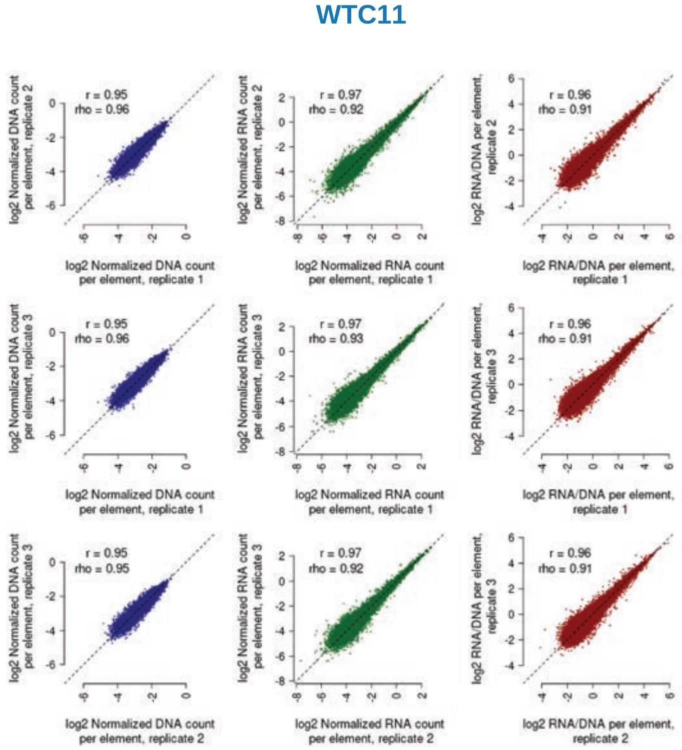
WTC11
r = 0.95
rho = 0.96
r = 0.97
rho = 0.92
r = 0.96
rho = 0.91
r = 0.95
rho = 0.96
r = 0.97
rho = 0.93
r = 0.96
rho = 0.91
r = 0.95
rho = 0.95
r = 0.97
rho = 0.92
r = 0.96
rho = 0.91
log2 Normalized DNA count per element, replicate 2
log2 Normalized RNA count per element, replicate 2
log2 RNA/DNA per element, replicate 2
log2 Normalized DNA count per element, replicate 3
log2 Normalized RNA count per element, replicate 3
log2 RNA/DNA per element, replicate 3
log2 Normalized DNA count per element, replicate 1
log2 Normalized RNA count per element, replicate 1
log2 RNA/DNA per element, replicate 1
log2 Normalized DNA count per element, replicate 2
log2 Normalized RNA count per element, replicate 2
log2 RNA/DNA per element, replicate 2

e

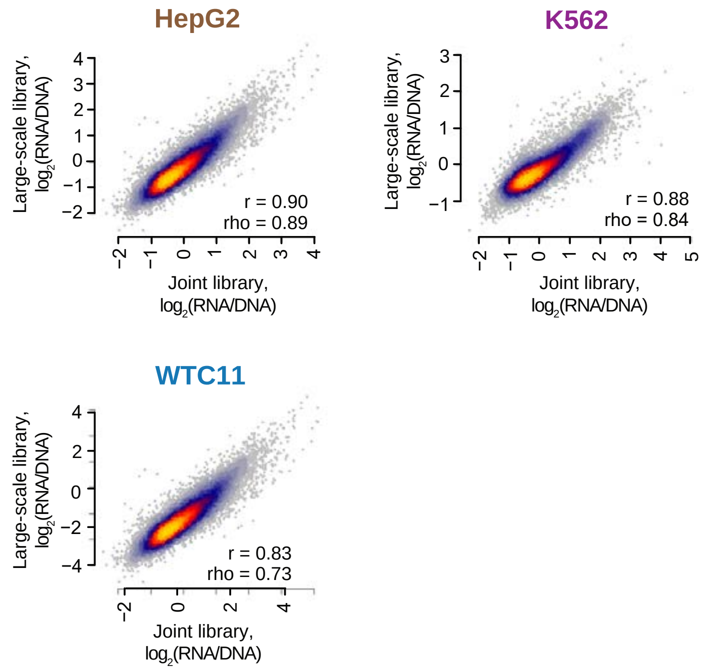
HepG2
K562
WTC11
Large-scale library, log2(RNA/DNA)
Joint library, log2(RNA/DNA)
r = 0.90
rho = 0.89
r = 0.88
rho = 0.84
r = 0.83
rho = 0.73

f

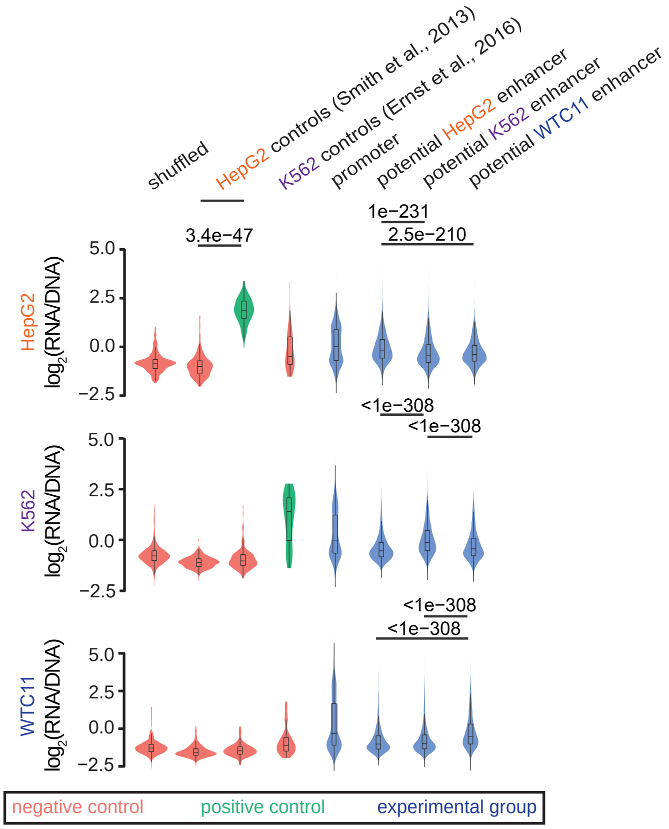
shuffled
HepG2 controls (Smith et al., 2013)
K562 controls (Ernst et al., 2016)
promoter
potential HepG2 enhancer
potential K562 enhancer
potential WTC11 enhancer
3.4e−47
1e−231
2.5e−210
<1e−308
<1e−308
<1e−308
<1e−308
HepG2 log2(RNA/DNA)
K562 log2(RNA/DNA)
WTC11 log2(RNA/DNA)
5.0
2.5
0.0
−2.5
negative control
positive control
experimental group

Supplementary Fig. 7: Quality control characteristics of the joint MPRA library. a, Histograms indicating the number of observed barcodes per element, for each of the three replicates and three cell types tested using the joint MPRA library. Shown with a vertical red line is the median number of barcodes per element. **b-d,** Scatter plots displaying the relationship between observed DNA counts (blue), RNA counts (green), and RNA/DNA ratios (red) for all pairwise comparisons among replicates, for the joint MPRA library tested in (**b**) HepG2, (**c**) K562, and (**d**) WTC11 cells. Candidate elements supported by fewer than 10 barcodes were filtered out prior to this analysis to reduce the impact of technical noise. **e,** Scatter plots displaying the relationships between activity scores for the subset of elements common to both the joint and large-scale MPRA libraries tested in HepG2, K562, and WTC11 cells. Also indicated are the Pearson (*r*) and Spearman (rho) correlation values. **f,** Violin plots of element activity [measured as $\log_2$(RNA/DNA)] in each of the three cell types for different element categories represented in the joint MPRA library shown in **Fig. 5a**. The difference between each pair of distributions tested was evaluated with a one-sided Wilcoxon rank-sum test, adjusted with a Bonferroni correction to account for the total number of hypothesis tests.

Supplementary Fig. 8

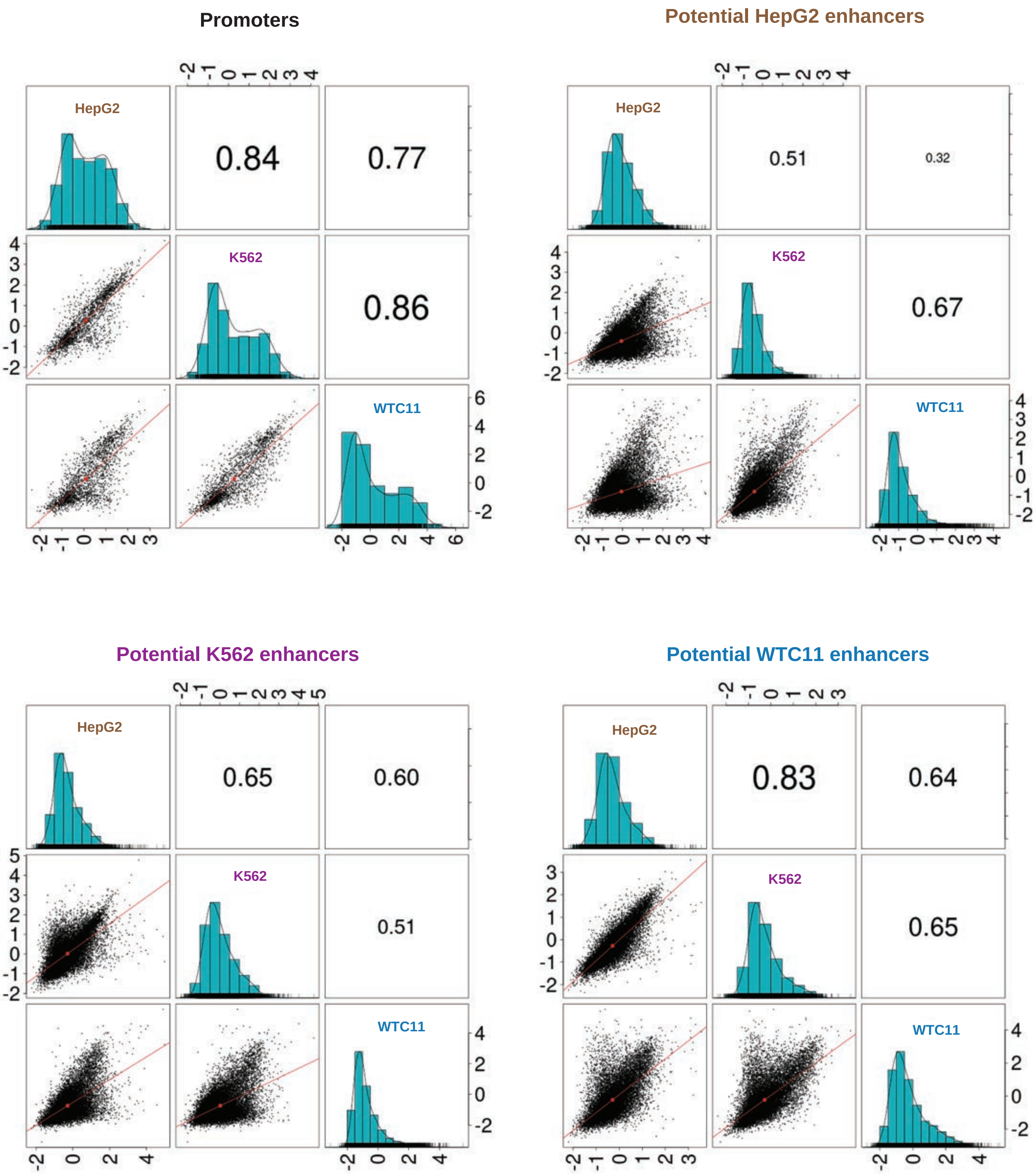

Supplementary Fig. 8: Comparison of promoter and enhancer activities from a joint MPRA library tested in three cell types. Scatter matrix displaying scatter plots corresponding to each of the three pairs of possible inter-cell-type comparisons (lower diagonal elements), for each of four element categories: i) protein-coding gene promoters, ii) potential enhancers selected from HepG2 cells, iii) potential enhancers selected from K562 cells, and iv) potential enhancers selected from WTC11 cells. Shown on the diagonal is a histogram of element activity scores [measured as $\log_2$(RNA/DNA)]. Also shown are Pearson correlation values among each pair of comparisons, with the size of the text proportional to the magnitude of the correlation coefficient (upper diagonal elements).

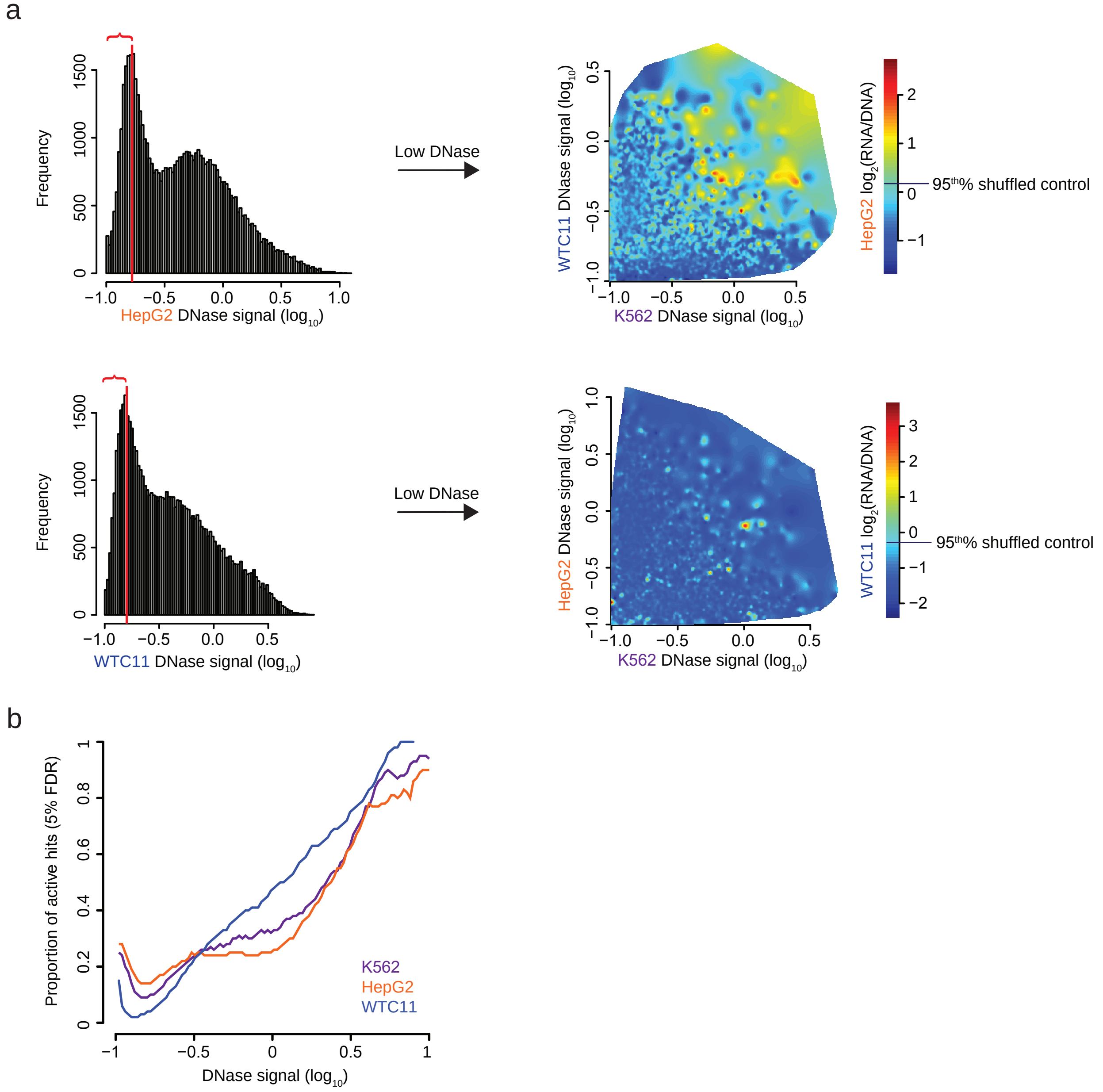
a
Frequency
HepG2 DNase signal (log10)
Low DNase
WTC11 DNase signal (log10)
K562 DNase signal (log10)
HepG2 log2(RNA/DNA)
95th% shuffled control
Frequency
WTC11 DNase signal (log10)
Low DNase
HepG2 DNase signal (log10)
K562 DNase signal (log10)
WTC11 log2(RNA/DNA)
95th% shuffled control
b
Proportion of active hits (5% FDR)
K562
HepG2
WTC11
DNase signal (log10)

Supplementary Fig. 9: Activity of elements with low DNase signal. a, In each of the depicted cell types, we quantified DNase signal and selected the subset of elements with low signal (*i.e.*, having a signal below the threshold indicated by the vertical red line). For this subset of elements, we evaluated DNase signal in the other two cell types and then quantified a smoothened kernel density estimate of MPRA activity in the cell type being examined. **b,** Point estimate of the proportion of active elements (*i.e.*, exceeding the signal for shuffled negative controls with a 5% FDR) as a function of DNase signal in each of three cell types. Each point along the x-axis reflects this proportion for elements with a DNase signal in a range of (x–0.1 to x).

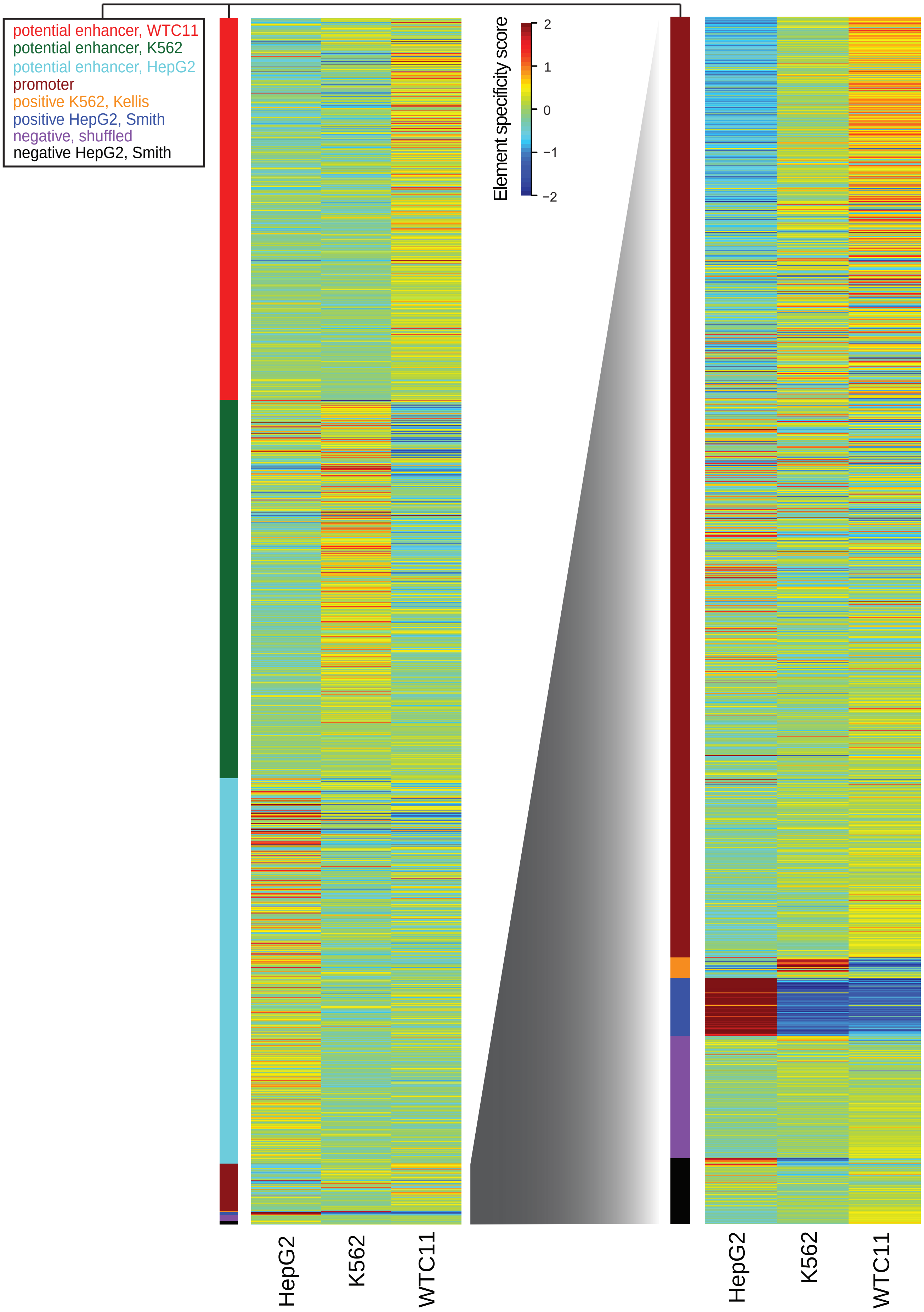
potential enhancer, WTC11
potential enhancer, K562
potential enhancer, HepG2
promoter
positive K562, Kellis
positive HepG2, Smith
negative, shuffled
negative HepG2, Smith
Element specificity score
2
1
0
−1
−2
HepG2
K562
WTC11
HepG2
K562
WTC11

Supplementary Fig. 10: Comparison of element specificity scores derived from a joint MPRA library tested in three cell types. Heatmap of element specificity scores (*i.e.*, computed as the deviation of an element's activity from its mean activity in all cell types). The heatmap shown on the right is a zoomed version of the heatmap on the left for elements other than potential enhancers. Elements are colored according to their category in the key provided.

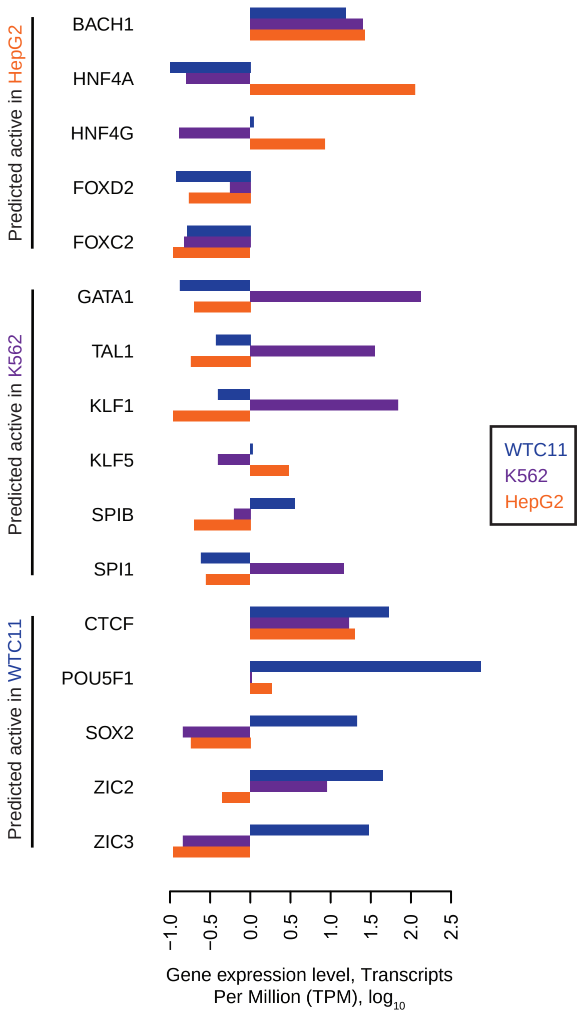

Predicted active in HepG2
BACH1
HNF4A
HNF4G
FOXD2
FOXC2
Predicted active in K562
GATA1
TAL1
KLF1
KLF5
SPIB
SPI1
Predicted active in WTC11
CTCF
POU5F1
SOX2
ZIC2
ZIC3
WTC11
K562
HepG2
−1.0
−0.5
0.0
0.5
1.0
1.5
2.0
2.5
Gene expression level, Transcripts Per Million (TPM), log10

Supplementary Fig. 11: Gene expression levels of TFs in each of the three cell types, for the members of TF families predicted to be highly active in each cell type based upon motif enrichment analysis. Shown are barplots indicating expression levels measured as Transcripts Per Million (TPM) from RNA-seq data derived from each of the three cell lines.
